# A Treatise on the Diseases of the Heart and Great Vessels

**Published:** 1832-04-01

**Authors:** 


A TREATISE ON THE DISEASES OF THE HEART AND GREAT VESSELS, COMPRISING A NEW VIEW OF THE PHYSIOLOGY OF THE HEART'S ACTION; ACCORDING TO WHICH THE PHYSICAL SIGNS ARE EXPLAINED. By *J. Hope*, M.D. formerly House Physician and House Surgeon to the Royal Infirmary of Edinburgh; Physician to the Mary-la-Bonne Infirmary, &c. Octavo, pp. 612. London, 1832.

IN our last number we had barely sufficient space to notice this work in a general manner, and advert to the opening chapter which treats of the relative anatomy of the heart. We have already said that we hail this as one of the many signs that surround us, of the progress of a rational and unprejudiced mode of investigation of disease in this country. When men of sense and science, young men too and rising ones, laugh at the obstinacy of bigots and the sneers of those who, standing still themselves, would have all others stand still also, when this is seen and done, the prospect is fair and encouraging for truth. But we shall confine ourselves, at present, to the subject immediately before us, and lay before our readers an abstract of those portions of Dr. Hope's volume, which we think will prove most generally useful and acceptable.

"The work is divided into six parts; I. The Anatomy and Physiology. II. Inflammatory affections. III. Organic affections. IV. Nervous affections. V. Miscellaneous affections. VI. Cases. Although every arrangement of diseases of the heart presents considerable difficulties, and I am by no means perfectly satisfied with the one which I have adopted, it appears to me preferable to others, because affections of the same class, being thrown together, by juxta position reflect light upon each other; nor, at the same time, are the inflammatory, and the organic affections in general so intimately connected, as to render their separation impossible without doing violence to the continuity of the subject. The miscellaneous affections are ranged by themselves, because they are not reducible to any of the preceding heads." xxv.

This is perhaps as useful an arrangement as any other; indeed we believe that provided arrangements are free from confusion, and are such as might obviously arise out of the subject, when its parts are pursued in natural order, we say that provided arrangements fulfil these indications they offer as much as can be fairly expected from them. Nosological refinements have this great fault, that they must necessarily be founded in some measure on theory, and this having no real and unchangeable existence in nature, varies with different men and ages, so that at last what was intended to give clearness and consistency to facts and opinions, serves but to perplex and confuse them. We have only to refer to many of the best writings of the last century for the truth of this remark. We beg to draw attention to another passage in our author's introduction, and express our regret that a similar course were not more generally and equally honestly pursued. We can give our testimony to the accuracy of the statement.

"As the authenticity of cases and observations is of the first importance, I deem it necessary to present a short explanation of the manner in which I have conducted my investigations. Being persuaded that no evidence is so suspicious as that of the senses, because the magnitude of an error is in proportion to the certitude which is supposed to attach to that mode of exploration, it has constantly been my endeavour to avail myself of the collective testimony of many. Accordingly, I have, for publication, preferred hospital cases, as being the best attested; I have invariably *written* the opinions or *diagnoses* before the death of the patient; have publicly tested them by the results of post-mortem examination; have minuted the dissections with the subject before me, and according to the prevailing opinion of the individuals present; and, generally before laying down my journal, I have annexed such remarks as the case suggested, while the circumstances were fresh in my recollection. Finally, I have obtained signatures where a case was very remarkable, or where there appeared a possibility of its being subsequently called in question. The cases appended to this work are nearly verbatim transcripts from journals thus kept; and, in order that they might present a just idea of the possibility of detecting disease of the heart, I have not taken them by selection, but, excepting a few, mostly without diagnoses, have introduced the whole of which I took notes in St. George's Hospital within a definite period. They will be found, I believe, to substantiate the view which I have offered of the heart's action—according to which the physical signs are explained; and, to the practical student of auscultation, by standing in the relation of exercises to a grammar, I entertain hopes that they may prove one of the most acceptable portions of the volume." xxvii.

Nothing could be more fair, more characteristic of a zealous love of truth than this method of study and of recording what is witnessed. Yet men must not be blinded even by this, nor take even evidence of so high a character for more than it is worth. Such is the construction of the human mind, that guard against error as we will, it steals on in our despite. Though many men agree upon a point, it is not necessarily true, nor is it necessarily false, though the majority vote it to be so. Those who see much of mixed investigations will probably agree with us in what we are about to state. Either one individual, from his talents, station, confidence, or other circumstances, gives the law to which the others bow, or seem to bow, or two or more parties are formed, who, finding they cannot agree, most commonly come at last to a compromise, which is usually in the favour of the most pertinacious and most patient. This is no caricature, nor is it said in jest. The history of medicine furnishes but too many instances in which majorities have gone on observing facts and warranting them, which have turned out at last to be false facts notwithstanding. Perhaps we may be playing the same game now, and our scrupulous accuracy may kindle a smile in the twentieth century. We do not make these observations to derogate in the smallest degree from the merit or the confidence due to Dr. Hope. He has taken the best mode which human ingenuity can devise, for procuring and ensuring the objects of truth, but still we must employ the means of judging and of weighing evidence with which Providence has endowed us, in order to render the attainment of those objects still more probable and easy.

Passing over the first chapter to which we have already alluded, we stop at the second, which treats of the action of the heart.

### ON THE ACTION OF THE HEART.

When the ear or a stethoscope is applied to the region of the heart, two successive sounds, followed by an interval of silence or repose, are distinctly heard. With the first sound, or at least so near it that the interval is scarcely if at all appreciable, there is usually a stroke or impulse communicated to the ear of the examiner, whilst the second sound is accompanied with little or none. The first sound is also duller than the second, slower, more prolonged, and terminating in it without a perceptible interval; the second is louder and smarter, "like the flapping of a bellows valve." Whoever applies his ear to the chest of a healthy person will distinctly hear the sounds, and feel the impulse of the heart, but it requires some practice to be enabled to analyse and discriminate them in the foregoing degree. These *sounds* were first noticed by Laennec. He attributed the first to the contraction of the ventricle, the second to that of the auricle. For eight or ten years this opinion of Laennec remained unquestioned, the diseases of the heart were explored stethoscopically with these views, and though some erroneous diagnoses were made, that is, published, other, and remarkably correct ones, were undoubtedly derived from them. This is of importance, in assisting to decide some questions that will presently arise. Well then, Mr. Turner called in question Laennec's exposition of the cardiac sounds, because the auricular contraction which he counted *second,* was found in the experiments of Haller, Harvey, Lancisi, &c. not to follow, but *precede* the ventricular, in fact to be the first. But Mr. Turner was more successful in shaking another's tree than in growing his own, and he was followed by Dr. Williams, who was yet more eminently unhappy in his conjectures, and by Dr. Corrigan who was very violent and very wrong. For the speculations of these gentlemen, and for arguments which we urged at the time against their views, and we presume with good effect, since they now appear to be generally abandoned, whereas at that time our contemporaries were "in most wicked haste to speed" to their adoption, for these and other matters connected with the subject we must refer our readers to former numbers of this Journal, and proceed to Dr. Hope's experiments and conclusions. His first experiments were performed on rabbits and small animals. Owing to the rapidity of their circulation much could not be proved, though one thing was so to demonstration:—that Dr. Corrigan and his colleagues are probably the only men in the world who could see what they saw, hear what they heard, and be satisfied of what they were satisfied of. As Dr. Hope's experiments were detailed at the time in the Medical Gazette, we cannot republish them here. At the conclusion of these experiments and researches Dr. H. entertained the following impressions, which we believe were also entertained by those who witnessed the experiments, and had previously paid any attention to the subject.

"That, in small animals, the auricular systole took place immediately *before* the ventricular, and not *after*, as supposed by Laennec, I regarded as certain, both from the evidence of my own experiments and from the concurrent testimony of the old physiologists. It was to be presumed that the same occurred in larger animals, but it remained to be proved.

That the *impulse* and *first sound* were referable to the ventricular, and not to the auricular contraction, I was equally persuaded, 1st, because the pulse, unquestionably a result of the ventricular systole, coincided so nearly, if not in every case perfectly, with the impulse and sound, that these three phenomena did not admit of being ascribed to any but the same cause; 2d, because clinical observations had proved to me, that certain anormal modifications of the heart's impulse and first sound corresponded with certain morbid conditions of the ventricular, but not of the auricular parietes.

That the *second sound* did not depend on the auricular systole, was indubitable; because the one was prior, and the other subsequent, to the ventricular contraction.

That it did not depend on the closure of the auriculo-ventricular valves was equally certain; because the closure of those valves takes place at the commencement of the ventricular contraction, whereas the sound occurs at its termination.

That it was not due to any other action of the auriculo-ventricular valves was obvious from physical considerations of their anatomical structure.

That it was not ascribable to the retrocession of the semilunar valves, I entertained a strong presumption, from having found the sound unimpaired, though the valves, on one side of the heart at least, were rigid with ossification; and the presumption amounted almost to certainty, from my having found the sound not only undiminished, but increased, in cases of enormous dilatation of both ventricles, in which it was impossible that the cavities could ever empty themselves; and where, consequently, the motion of the valves must have been impeded by the constant pressure of fluid on both sides (vid. for instance, case x.)" 17.

It remained to be ascertained what was the cause of the second sound, and, we may add, that it remained to be more conclusively established that the ventricular systole was the cause of the first. Not to enter into reasonings too discursive for the pages of a review, Dr. Hope was led to conjecture that the sound was owing, in some manner, to the ventricle, and that it was produced by the influx of the blood into the ventricle after its contraction. In order to determine this, if susceptible of determination, our author resolved to perform some experiments on larger animals, in which the results would be less unsatisfactory. Many gentlemen were present, during their performance, at the veterinary institution of Mr. Field, in Oxford Street, and we can vouch for their having been conducted in a manner as little calculated as might be to vitiate the conclusions, or favour error. Before proceeding to the experiments, the following queries were distributed to those present, as well as sent to Mr. Brodie, who was prevented from attending in person.

"1. Do the auricles contract immediately before the ventricles?
2. Does an interval occur between the two contractions, or is the succession so rapid as to amount to continuity of action?
3. Does the ventricular contraction cause the impulse, pulse, and first sound?
4. Do the ventricles contract completely, and do they remain closed and empty, during the interval of repose? Or——
5. Do the ventricles dilate again immediately after their systole; and is this dilatation attended with an influx of blood from the auricles?
6. Is the influx of blood into the ventricles during their diastole the cause of the second sound? If not——
7. What is the cause of the second sound?" 21.

Perhaps it may be thought that we are dedicating too much space to physiological and experimental inquiries; but the fact is, that the whole question hinges, as Lord Castlereagh would say, on these fundamental premises. Unless the correspondence of the sounds with certain physiological conditions of the heart is established, where are we to look for diagnosis through the medium of auscultation? At the same time we shall endeavour to be as brief as possible, and rather indicate the results than the means of arriving at them. For these we must refer to the work itself. Rabbits, then, having been found to be too small to furnish satisfactory data, asses were selected, and as many as five experiments were made, in the presence of several physicians and surgeons of character and judgment. In order to shew the manner in which the experiment was conducted, we may select the following passage.

"EXPERIMENT II. The heart of an ass was exposed to view in the same manner as before, but with still greater celerity. For *about a minute only*, the action was quivering and irregular; it then fell to its natural standard (40 to 50 per minute), became *perfectly regular*, and the ventricular contraction, as felt by the hand and the stethoscope, was performed with a power which can scarcely be imagined from an examination on the outside of the chest.

Three successive motions—namely, the auricular systole, the ventricular systole, and the ventricular diastole—were now distinctly recognized and acknowledged by all who witnessed them. The stethoscope was applied to the ventricle, and the *two* sounds were clearly and unequivocally heard even by those who were unaccustomed to the instrument. Five gentlemen listened deliberately twice over, and two of them, three times, before it was dictated that, 1st, *Drs. Hewett and Hope, and Messrs. Lane, Field, and Cooper, listened successively through the stethoscope applied to the ventricle, and severally counted* 1, 2, *synchronously with the sounds which they heard; while the others ascertained, by the touch and sight, that the sound* 1 *coincided with the ventricular systole, and the sound* 2 *with its diastole.*

This part of the experiment was so deliberately performed that it occupied from ten minutes to a quarter of an hour, as near as could be judged from the whole time expended (from twenty to twenty-five minutes), and each of the experimenters was asked whether he was satisfied, whilst he had still an opportunity of renewing his examination.

It was now submitted to investigation, how the ventricular systole could occasion the impulse; since the body of the organ appeared to *recede* during that motion. The result was the following note:

2. *While the ear rested on the stethoscope applied to the middle of the ventricle, the impulse was felt by the auscultator to coincide with the systole, notwithstanding that the body of the ventricle appeared to be receding at the moment the impulse took place.*

During the course of the experiment the action of the auricle was again examined. Its anterior edge and surface only were in sight, the root and sinus being concealed behind the ventricle. It was noted that,

3. *The auricle never emptied itself, and its contraction was always very inconsiderable. The anterior edge and surface were seen to retract with a rather sudden motion; but as the extent of the motion was very inconsiderable, it had the appearance of being feeble.*

The contraction of the auricle was so much less than there was reason to anticipate from the extent of its action in smaller animals, that it was questioned whether it was, in the present instance, performed with the natural vigour. The extraordinary power with which the ventricle acted, favoured the affirmative; and as the proportion of the auricle to the ventricle is singularly less in large animals than in small, there is reason to suspect that they perform a less important function in the former." 28.

The results may be comprehended in the ensuing quotation. We think, however, that Dr. Hope has not arranged these experiments with the conclusions deducible from them, so lucidly as he might have done. Had he merely stated the facts observed in the experiments first, and subsequently the inferences or conclusions to which those facts might legitimately appear to give rise, he would have avoided what appears to us to savour a little of confusion and iteration. When the work shall come, as we have no doubt that it will, to another edition, Dr. H. may perhaps be enabled to make some alterations in the arrangements of this portion of it.

"*Of the Motions of the Heart.*

1. The auricles contract so immediately before the ventricles, that the one motion is propagated into the other, almost as if by continuity of action; yet the motion is not so quick that it cannot readily be traced with the eye.
2. The extent of the auricular contraction is very inconsiderable, probably not amounting to one-third of its volume. Hence the quantity of blood expelled by it into the ventricle, is much less than its capacity would indicate.

3. The ventricular contraction is the cause of the impulse against the side ; first, because the auricular contraction is too inconsiderable to be capable of producing it; second, because the impulse occurs after the auricular contraction, and simultaneously with the ventricular, as ascertained by the sight and touch ; third, because the impulse coincides with the pulse so accurately as not to admit of being ascribed to any but the same cause.
4. It is the apex of the heart which strikes the ribs.
5. The ventricular contraction commences suddenly, but it is prolonged until an instant before the second sound, which instant is occupied by the ventricular diastole.
6. The ventricles do not appear ever to empty themselves completely.
7. The systole is followed by a diastole, which is an instantaneous motion, accompanied with an influx of blood from the auricles, by which the ventricles re-expand, but the apex collapses and retires from the side.
8. After the diastole, the ventricles remain quiescent, and in a state of apparently natural fulness, until again stimulated by the succeeding auricular contraction.

*Of the Sounds.*

9. The *first sound* is caused by the systole of the ventricles.
10. The *second sound* is occasioned by the diastole of the ventricles.

*Of the Rhythm.*

Order of succession.

1. The auricular systole.
2. The ventricular systole, the impulse, and the pulse.
3. The ventricular diastole.
4. The interval of ventricular repose, towards the termination of which the auricular systole takes place.

*Duration.*

This is the same as indicated by Laennec, viz.
The ventricular systole occupies half the time, or thereabout, of a whole beat.
The ventricular diastole occupies one-fourth, or at most one-third.
The interval of repose occupies one-fourth, or rather less.
The auricular systole occupies a portion of the interval of repose." 30.

We were present at the performance of some of the experiments, and can, therefore, vouch for the accuracy with which they are detailed, as well as for the unanimity which prevailed in all who witnessed them, respecting the conclusions to be drawn from them. It appears to be undeniable that the first sound, the impulse, and the pulse, coincide with the ventricular systole ; that the second sound is connected with the ventricle, perhaps dependent on its diastole, perhaps not, but ventricular certainly ; and, lastly, that the auricle has nothing to do with either sounds or impulse, nay, that it does not seem to act with sufficient regularity to offer a probability of any regular or constant sign attending its actions, natural or morbid. Now the establishment of the close and inseparable connexion of the cardiac sounds and the impulse with ventriclar action, and especially of the first sound, the impulse, and the pulse with ventricular contraction, is of paramount importance. In the investigation of disease, we generally find the alterations of sound chiefly noticeable in the first, and in it the different bruissemens are commonly perceived. They indicate, they have always seemed to indicate, alterations in the ventricular constitution—they are found by post-mortem investigations to do so—and, if specious reasonings or deceptive experiments had led men to believe that they were not connected, but that the first sound was auricular and not ventricular, our facts would have been vitiated, our experience confounded; in short, the auscultic study of the heart would have been based in worse than ignorance, in positive error. It was from experience, from the consciousness of the first sound and impulse having proved, in practice, so safe a guide in studying ventricular alterations, that we all along opposed the heresies and fallacies of gentlemen, to whom we have sufficiently alluded already.

Dr. Hope makes many interesting, indeed valuable, remarks upon the action of the heart, and of its component parts, but having given the pith of his conclusions, and having cleared our way of some outstanding errors and misconceptions, we must refer to the work itself for further information on this subject. We have stated enough to enable the auscultator to proceed with confidence, and the public to receive his deductions without misgiving. At the same time we must make one observation which appears to us to be warranted by the present bearings of this important question. Although we have no doubt that the first sound, impulse, ventricular systole, and pulse coincide, and that the second sound is produced by the ventricle, yet we do not consider the manner in which the latter is produced as exactly determined, and all is yet uncertainty with respect to the physical signs dependent on the auricles, if any do depend upon them: nay, we are not even accurately informed of the laws which regulate and rhythm which characterises their ordinary contractions. We press these remarks the more strongly because in the investigation of disease we have long been struck with the uncertainty which attends the diagnosis of auricular alterations, and as their physical signs were always looked for in the phenomena developed by the second sound, it can occasion no surprise that such should be the case. We must remember that at present then we possess no *auscultic* means of determining the condition of this portion of the cardiac organ. It is better at once to own a truth, than to attempt to delude ourselves or mystify others.

The second section which treats of the physiological phenomena of the heart's action must be passed over. As our space is necessarily limited we must confine ourselves to the more strictly practical parts of the volume. What has hitherto occupied us was absolutely necessary, because it was and is the foundation of practical diagnosis. Neither can we devote much space to the pathological phenomena of the heart's action, for these admit of being noticed in the description of its particular affections. However, there are some points that we cannot entirely neglect.

1. In hypertrophy the first sound appears to be prolonged, and the impulse is augmented and frequently lengthened. The second sound is frequently diminished, sometimes almost obliterated as it were by the preponderance of the first. These are phenomena that will strike every careful observer, and we subjoin Dr. Hope's explanation of them. "The power of the impulse," says he, "is increased in the direct ratio of the hypertrophy; and the movement is a progressive heaving, because the hypertrophous ventricle *contracts slowly*, and with a gradual progression. For the same reason the first sound is diminished—the impulse communicated to the contained fluid not being sufficiently smart to occasion more than a dull, stifled sound, if any at all. The second sound is dull and weak because the unyielding thickness of the ventricular walls renders their expansion by the fluid more difficult and gradual;—consequently, the re-action, generating the sound, is more languid."

In ventricular dilatation the impulse is slight, brief, the first sound clear and loud, the second also increased. The reasons for these circumstances may be found in the converse of their causes and attendant phenomena to those of hypertrophy. We must confess that we have found the physical signs of dilatation less conclusive and satisfactory than those of hypertrophy, and that we have made more mistakes and seen more made with the former than with the latter. Were auscultic signs trusted to alone we believe that the stethoscopist would be wrong as frequently as right, with respect to moderate dilatation of the ventricles. But the judicious practitioner is he who combines all means of diagnosis —general symptoms, special signs, the knowledge derived from cadaveric investigations, and the calculation of probabilities from mixed or doubtful evidence: who strengthens the weak part of one means of diagnosis with the helps derivable from another.

In hypertrophy with dilatation the sounds produced are a mixture of those occasioned by either. In valvular disease certain murmurs are produced, but these may be noticed more particularly hereafter.

"Obstructions of a hard, rugged kind, as ossifications, occasion louder murmurs than smooth obstructions, because they more completely break the current of the blood. Murmurs are not, as is often supposed, louder, cæteris paribus, in proportion as the valvular contraction is greater. On the contrary, the loudest murmurs are produced by a moderate contraction, and they become weak when it is extreme. Thus, a rugged osseous concretion, the size of an ordinary pea, in the aortic orifice, I have found to produce the loudest possible murmur; whereas, a contraction of the mitral or tricuspid valve to the size of only two, three, or four lines in diameter, I have frequently known to occasion little or no murmur. Osseous asperity, alone, without contraction, produces considerable murmur. From the above premises, it may be stated as a general principle, that the loudness of a murmur is in proportion, not only to the roughness of the obstacle, but also to the quantity of fluid transmitted through the valve and put in preternatural commotion by the obstacle. The effect we should naturally expect to be aided by the force and velocity with which the fluid is impelled; and, accordingly, we find that when the ventricle is hypertrophous, or the circulation hurried, the murmur is proportionably louder. A simple illustration of these doctrines may be obtained by employing air instead of fluid, as the sonorous medium. Thus, if air be blown with equal velocity through a large, and a small orifice or tube, the sound is louder from the former. If the velocity be increased, the sound is proportionably augmented, and it is, cæteris paribus, always louder when the tube or orifice is rough or unequal.

A contraction of the mitral or tricuspid orifice to the size of the natural aortic or pulmonic orifice, generally produces a murmur. On what does this depend? Not on the mere circumstance of the contraction; for the aperture, being still as wide as that which affords egress to the blood, ought, therefore, to be equally capable of admitting the fluid without murmur. The murmur depends, I imagine, on the different configuration and position of the arterial, and the auriculo-ventricular orifices. The latter, instead of opening into tubes of their own dimensions, as in the case of the aortic and pulmonary orifices, open, generally with more or less infundibuliform projection, into spacious cavities: and, as the blood, on its delivery, must diverge to fill these cavities, it forms, in this act, various whirls, eddies, and counter-currents, which I conceive to be the cause of the sound. When the contracted valve, instead of projecting into the ventricle, is stretched tense across its orifice, the murmur is proportionably louder. This probably depends on the hydraulic law, that fluids escape with a more abundant and tranquil stream from a convergent pipe, than from a simple perforation of the same dimensions, through a plane surface. It is, perhaps, partly for an analogous reason that a *slight* patescence of the mitral or tricuspid valve occasions, by regurgitation, a louder sound than might be anticipated from the smallness of the aperture; for, in this case, from the construction of the valve, the discharging orifice projects into the fluid to be discharged,—a circumstance the most adverse of all others to the free and tranquil delivery of the fluid. This, however, is not the only reason for the loudness of the murmur. Another is, that, in consequence of the superior power of the ventricular contraction, the blood, propelled retrograde by it, moves with greater force and velocity, than it flows forwards by its natural moving powers.

A slight patescence of the valve admitting of regurgitation, may result from a structural lesion not sufficient to present an obstacle to the blood flowing in its natural direction from the auricle into the ventricle,—as that, for instance, from a contraction of the chordæ tendineæ, preventing the margins of the valve from coming in perfect apposition; and in this way is to be explained a phenomenon which I have frequently noticed; namely, that a murmur from regurgitation sometimes accompanies the first sound, when none attends the second. A slight contraction, indeed, such as, for example, to diminish the circumference by a quarter, or from that to half an inch, does not, unless accompanied with ruggedness, occasion any appreciable murmur with the second sound; for the blood has little sufficient space to pass with tranquillity; but if, at the same time, the ventricle is dilated and hypertrophous, a considerable murmur may be created; for the increase of the quantity and velocity of the blood which enters from the auricle, produces the same effect as a greater degree of contraction of the orifice." 62.

Murmurs in cases of hypertrophy with dilatation are produced by the greater capacity and alteration of form of the ventricle in relation to the artery which issues from it. The section on murmur of the heart and arteries independent of organic disease is well worthy of attention, and tends very far to the elucidation of these litigated points. Those who have studied Laennec's work are aware that he has been led into inconsistencies, if not absolute absurdities on the subject of the bellows sound and the arterial thrill (frémissement cataire), independent of organic disease. Indeed Laennec is hardly himself when he arrives at the study of cardiac disease; the two portions of his work, that on the lungs and that on the heart, are like the feet of Nebuchadnezzar's image, of iron and clay—they cleave but do not amalgamate. When we come to investigate for ourselves in practice we do not find Laennec's statements on many points, and more particularly of the nervous bellows sound and thrill in the arteries, satisfactorily borne out. As we said above we think Dr. Hope's remarks on this subject particularly good.

"As my own experience does not accord with that of Laennec as to which motions of the heart are accompanied by the murmur, it is necessary to premise that I have found it accompany the *systole* of the ventricles; and not the diastole, unless it, at the same time, and in a predominant degree, attended the systole. In the arteries, it coincides with their diastole. The purring tremor occurs at the same moment and is a result of the same cause. The arterial thrill is nothing more than a less degree of the purring tremor."

Dr. H. believes that the murmurs and tremors, both in the heart and in the arteries are occasioned by modifications in the motion of the fluid. He brings forward conclusive evidence to prove that the motion of fluids in tubes occasions sound, independent of the presence of air, and modifications in the sound are occasioned by modifications in the tube. To show that when the sounds in the heart and arteries do occur there is an increase of friction dependent on a modification of the motion of the blood to account for them, he brings forward the following important facts.

"Eight or ten dogs were blooded more or less frequently, from once to ten or twelve times, and at intervals varying from twenty-four to seventy-two hours. The results were, that, on the day following the first or second abstraction of blood to the amount of eight or ten ounces, the systolic sound of the heart, previously loud and clear, became attended with a whizzing or sawing murmur,* the impulse increased and became unusually smart or abrupt, and the pulse became quick and jerking, with a thrill and a throbbing, perceptible over the whole body. These phenomena increased up to the fourth or fifth bleeding, when they appeared to attain their maximum, the sawing sound being extremely loud, the impulse and pulse violently jerking and bounding, the arterial thrill or purring tremor excessive, and the throbbing perceptible not only when the finger was placed on an artery, but when the hand grasped a large surface of the body. A hissing bellows-murmur was, moreover, distinctly heard, when the stethoscope was placed over any considerable artery, as the femoral or carotid. The pulse at this time generally beat from 150 to 190 per minute, its natural standard being about 120.

The phenomena underwent the following changes in correspondence with changes

* "These, it will be understood, are merely varieties of the *bellows-murmur*,—a term which may be taken in a generic sense."

in the circumstances. The animals being extremely nervous and irritable, the pulse was instantly accelerated ten or fifteen beats per minute by the slightest excitement, as that of being moved or startled; and the murmur and jerk sustained, in consequence, a remarkable increase.

After reiterated venesections the pulse became small and weak ; but, so long as it remained jerking (or *sharp*, as some would now designate it), the murmur continued, though not so loud as previously.

If venesection was omitted for three or four days, reaction subsided ; and in proportion as the pulse and impulse became softer, though without a loss of *real* strength and fulness, the murmur, both of the heart and arteries, the purring tremor, the general throbbing, and the nervous irritability, gradually disappeared.

If, during the full prevalence of all the phenomena, the animal was bled to the approach of syncope, the pulse and beats of the heart, reduced to about 100 per minute, became feeble and soft, and at the same time lost all murmur and thrill; but, in the course of from fifteen to thirty minutes, reaction was established and all the symptoms recurred.

If the animal was held erect by the fore legs, a posture which, either by diminishing the afflux of blood to the brain, or by obstructing the circulation through the heart and lungs, caused the gradual supervention of syncope, the pulse became slow, soft and feeble, and the murmur and thrill were suspended; but they were promptly restored to their former state when the animal was placed on its legs." 74.

From these experiments, it may be concluded that murmur and tremors are dependent on the spasmodic abruptness of the heart's action, that is, on the velocity with which the blood is propelled. Thus we find the bellows-murmur in those affections in which this condition exists ; in pericarditis—in those who labour under reaction from excessive loss of blood—and still more in the irritable and excitable of both sexes. In the latter, a very slight cause will excite this morbid action, a sudden movement, trifling exertion, an emotion, nay, an idea. Dr. H. has once seen death ensue from purely nervous action of the heart. The individual was a healthy young female, who became affected on the intelligence that her husband had deserted her, lay twenty-four hours in a state of almost complete insensibility, and the violently-bounding, jerking, and thrilling, arterial throb, together with universal flushing heat and perspiration of the surface, resisted every remedy, and only subsided with the wane of life.

"A few apparent anomalies remain to be explained. One portion of an artery, as the abdominal aorta, or the radial in a case of whitlow, may pulsate more strongly than another. The most simple and generally received explanation of this, is, that arteries are irritable parts, which, under the circumstances alluded to, and under many others, as blushing, increased local determination for the purpose of nutrition and secretion—exemplified by the state of the uterus and mamma during pregnancy, become *relaxed* or lose a portion of that tonic power by which they resist and support the distensive force of the heart's contractions. Consequently, as liquids flow most readily in that direction which offers the least resistance, the relaxed vessel admits an increased quantity of blood at each contraction of the heart; hence its throb,—which is a passive—not an active movement; and, as the augmented afflux is necessarily accompanied with increased velocity of the current, at least until the vessels become gorged, the result is an increase of friction, producing murmur. Another circumstance contributes to the production of the murmur: the relaxed artery, by yielding to the pressure of the heart, becomes not only distended, but elongated and tortuous. Hence, according to the principle developed in page 70, the friction and murmur are increased. One of the most tortuous arteries is the placental during gestation, and in none is the bellows-murmur more distinct. Some have imagined that the pulsation, in these cases, is quicker than than that of the heart. It is scarcely necessary to say that this is an error. Corresponding arteries in different limbs have been said to beat alternately stronger than each other,—to me, when I could persuade myself of the fact, it has always appeared to depend on differences of posture, by which the free afflux of blood to the one limb or the other, was prevented. In all circumstances, however, the murmur and thrill, according to the explanation which I have offered, depend on the motion of the fluid." 79.

We must recollect that the theory respecting the local relaxation of arteries, and their loss of a portion of their tonic power, is but a theory after all. Certain it is, that if arteries be dilated and the heart hypertrophic, if a large artery be diverted from its course, and encroached on in any degree, nay, if an artery be diseased, aneurismal in its course, it will furnish the peculiar thrill which has been the subject of the present section. Whatever makes the blood pass with more velocity and force, accompanied, perhaps, in many instances, with some alteration of condition in the contractile tissue of the vessel itself, seems capable of exciting the phenomenon. Dr. Hope remarks that the purely inflammatory pulse is unattended with thrill. This must depend on circumstances. In pericarditis, the arteries not unfrequently evince it.

### INFLAMMATORY AFFECTIONS OF THE HEART AND GREAT VESSELS.

Inflammation of the heart and pericardium has latterly received attentive and merited consideration. From its connexion with rheumatism, and its consequent frequency, it becomes an object of the utmost importance to be enabled to discriminate it without difficulty, and to determine the treatment most sanctioned by principle and successful in practice. The morbid changes consequent on carditis and pericarditis have been so accurately described by Baillie, and so fully by Latham, that we must pass over the chapters in the present volume which are devoted to their enumeration. We should mention that Dr. Hope treats of the inflammatory affections of the heart and great vessels in the following order: pericarditis—carditis—and, lastly, inflammation of the lining membrane of the heart and arteries. The diagnosis of pericarditis has been universally, or almost universally, considered as extremely difficult. With the aid of our modern knowledge respecting rheumatism, and the assistance derivable from physical means of diagnosis, that difficulty is greatly diminished. Dr. H. thinks that pericarditis may be nearly always detected, and we think that it may.

Passing over a very good account of the general symptoms of pericarditis, we stop at Dr. Hope's description of the physical signs.

"*The impulse* of the heart is greatly increased:—not only heaving the thoracic walls vigorously, but being remarkable for its abrupt or jerking character: whence it often shakes the whole anterior chest. Some beats are generally stronger than others, even when the action is regular. The pulse or rather throb of the arteries, often perceptible over the whole body, is of a corresponding nature, each undulation of the blood shooting with instantaneous velocity under the finger, as if through a lax or imperfectly-filled tube, and constituting what is called a bounding, or, more expressively, a jerking pulse,—the pulse that we feel during reaction after uterine or other excessive hemorrhage. Very frequently it is accompanied with a distinct thrill. Sometimes it is stronger and more voluminous, at others, smaller and weaker; yet, in the latter case, it still retains the same jerking character.

When the action of the heart becomes feeble and faltering, the impulse of the organ of course sustains a corresponding change; but, notwithstanding, the jerk accompanies any isolated contraction that happens to be strong.

An impulse and pulse of the jerking description denote an inordinately abrupt and as it were spasmodic, contraction of the heart, probably attributable to an increase of irritability resulting from the inflammation. It is this peculiarity in the beat, which distinguishes it from the beat of a merely accelerated circulation, a distinction perfectly familiar to practical men. The peculiarity subsists not only during the continuance of inflammation, but so long as the action of the heart remains quick after the inflammation has apparently subsided,—a period which generally occupies several weeks, and, if adhesion of the pericardium has taken place, frequently as many months. I have known it exceed half a year. In very protracted cases, it is probable that the irritability of the heart is kept up either by an occasional recurrence of inflammatory action, or by the unnatural circumstances in which the organ is placed by adhesion, or, finally, by a softened state of the muscular substance, the result of carditis.

*The Sounds.*—The sound of the ventricular systole is not only unusually sonorous, but is accompanied with a bellows-murmur. This sign was first noticed by Dr. Latham, who pointed it out to me at St. Bartholomew's Hospital in 1826. Since that time, I have never found it absent *when the heart presented the increased, jerking impulse* above described. Dr. Latham restricts his observation to rheumatic pericarditis; to myself the phenomenon has appeared to exist equally in every form of the disease. When the action of the heart has been feeble and faultering, I have found the murmur absent; but when, in the same case, the action has, either previously or subsequently, been strong and jerking, the murmur has existed. The reason of this will be obvious from the explanation which will presently be offered. The murmur sometimes continues after the heart appears to have resumed its natural action and the patient to be well; but, so long as it remains, as remarked by Dr. Latham,* 'his return to the habits and exertions of health will bring back palpitation and other symptoms, which bespeak the certainty of mischief still abiding in the heart.'

Not the ventricular systole only, but occasionally, though by no means always, its diastole likewise, is attended with the bellows-murmur; and I have found this supersede and, as it were, annihilate the natural second sound more completely in pericarditis, than, I think, in any other affection of the heart. Sometimes, in short, it is a pure whizzing, equally prolonged as, and almost continued into, the first sound." 111.

We beg to confirm the statements of Drs. Latham and Hope, respecting the presence of the bellows sound in cases of pericarditis. In nearly every case of the disease which we have had an opportunity of witnessing and examining, this sound has been present in a greater or lesser degree. As the accuracy of the opinion has been questioned, it is important for all who have had an opportunity of studying cases to record the results of their observation.

When the pericardium contains much fluid, the resonance of the præcordial region becomes dull over a greater space than natural; the impulse is undulatory, and not exactly coincident with the first sound. M. Louis states that he has found a temporary effusion of fluid cause a prominence of the cardiac region. Chronic pericarditis is undoubtedly more obscure than the acute form. The following short extract presents all that our author has to say on its diagnosis.

"*General Signs.*—The signs of chronic pericarditis are much the same as those of acute, but in a very inferior degree. The fever is more that of hectic or marcor, with occasional exacerbation when perhaps the inflammation becomes subacute. The anxiety and restlessness, though sometimes great, are comparatively supportable. The position is less constrained and I have observed that the patient often prefers the sitting posture, with the body inclined forwards. The circulation is less embarrassed, and the action of the heart, though often abrupt and jerking, is usually somewhat feeble, except during any temporary exacerbation of inflammatory action. The pulse, also, is sometimes not very unsteady, though the pericardium be full of fluid, which I attribute to the elasticity of the membrane not being so far destroyed by the inflammation as to prevent it from gradually undergoing extension, and accommodating itself to its contents. Whence compression of the heart by the fluid is in some degree obviated. The patient, I have thought, more frequently complains of a load and fulness, 'something which he cannot get down,' in the scrobiculus cordis, in chronic, than in acute pericarditis.

Chronic pericarditis, especially if such from its commencement, is more obscure than acute. I have, in former years, seen it overlooked more than once. But these cases, when I now revert to them, appear to me to have presented sufficiently cha-

---

* Lond. Med. Gaz. vol. iii. p. 214.

racteristic symptoms. The history affords the greatest light. If the patient, previously exempt from disease of the heart, has suddenly become affected with its symptoms, attended by marcor and some degree of fever, within a period seldom extending beyond a few months, and which he often dates from a blow or fall on the breast, a rheumatic fever, or an inflammation with pain in the præcordial region, chronic pericarditis may be strongly presumed; and if these symptoms coincide with the physical signs of fluid in the pericardium, the existence of the malady may be regarded as almost certain.

*Physical Signs.*—The impulse and pulse have much the same general characters as in acute pericarditis, except that, as the heart's action is less vigorous, they are not so strong. The sounds will vary according to circumstances. When the action of the heart is jerking and not wholly devoid of force, the first sound will be attended with a murmur, which, however, is generally very slight. When there is inflammatory constriction of the orifices, a murmur will attend both sounds. Should the heart be dilated, as is frequently the case, the sounds will be increased; and should hypertrophy be conjoined with the dilatation, the impulse will sustain a corresponding augmentation of force. The signs of fluid in the pericardium are the same as in acute pericarditis; namely, the extensive dulness on percussion, and the undulatory impulse." 115.

The chapter on the treatment of pericarditis is judicious, but need not detain us. The author very strongly and very properly insists on the powers of mercury. The fourth section is on adhesion of the pericardium, and as the frequency of this condition does not appear to us to be justly appreciated by practitioners in general, we shall devote a page or two to its consideration. It is a very common consequence of pericarditis, both acute and chronic, and especially of the rheumatic form. It almost always induces enlargement of the heart; indeed Dr. H. has never examined a case of complete adhesion without discovering the latter. We remember two instances of complete adhesion which we saw, and in which the heart appeared perfectly natural. It must be owned that these are rare exceptions to a rule even more than generally true. Our readers can have little difficulty in conceiving why such adhesions should be likely to induce such disease of the heart.

We had intended to have inserted here the signs considered by Dr. Hope as pathognomonic of adhesion of the pericardium. But for these and other matters we must refer to our next Number, the press of matter on the subject of cholera compelling us to bring this article to an abrupt conclusion.

